# *N*-(2,6-Diisopropyl­phen­yl)-*N*-{3-[(2,6-diisopropyl­phen­yl)imino]­butan-2-yl}aza­nide trichloridostannate(II)

**DOI:** 10.1107/S1600536812014729

**Published:** 2012-05-02

**Authors:** Xiaoli Ma, Shuai Sun, Pengfei Hao, Ying Yang, Zhi Yang

**Affiliations:** a School of Chemical Engineering and Environment, Beijing Institue of Technology, Beijing 100081, People’s Republic of China; b School of Chemistry and Chemical Engineering, Central South University, Chang Sha, Hunan, 410083, People’s Republic of China

## Abstract

In the title compound, (C_28_H_43_N_2_)[SnCl_3_], two pairs of molecular species are present in the asymmetric unit. The employed α-diimine opens up, forming a highly asymmetric ammonium that has its protons at one of the N atoms [N—C= 1.264 (4) and 1.516 (4) Å]. One of the C=N double bonds was oxidized to C—N, which is consistent with the bond length of 1.516 (4) Å. Meanwhile Sn^IV^ was reduced to Sn^II^. The (SnCl)_3_^−^ anion is trigonal–pyramidal. In the crystal, mol­ecules are linked by C—H⋯Cl, N—H⋯Cl, N—H⋯N and C—H⋯N bonds. The crystal studied was twinned by pseudo-merohedry.

## Related literature

For related α-diimine ligand complexes, see: Rake *et al.* (2001 ▶); Hinchliffe *et al.* (2007 ▶); Baker *et al.* (2008 ▶); Yang *et al.* (2010 ▶); Gao *et al.* (2011 ▶); Liu *et al.* (2011 ▶). For similar ionic complexes, see: Hill & Hitchcock (2002 ▶); Nie *et al.* (2010 ▶).
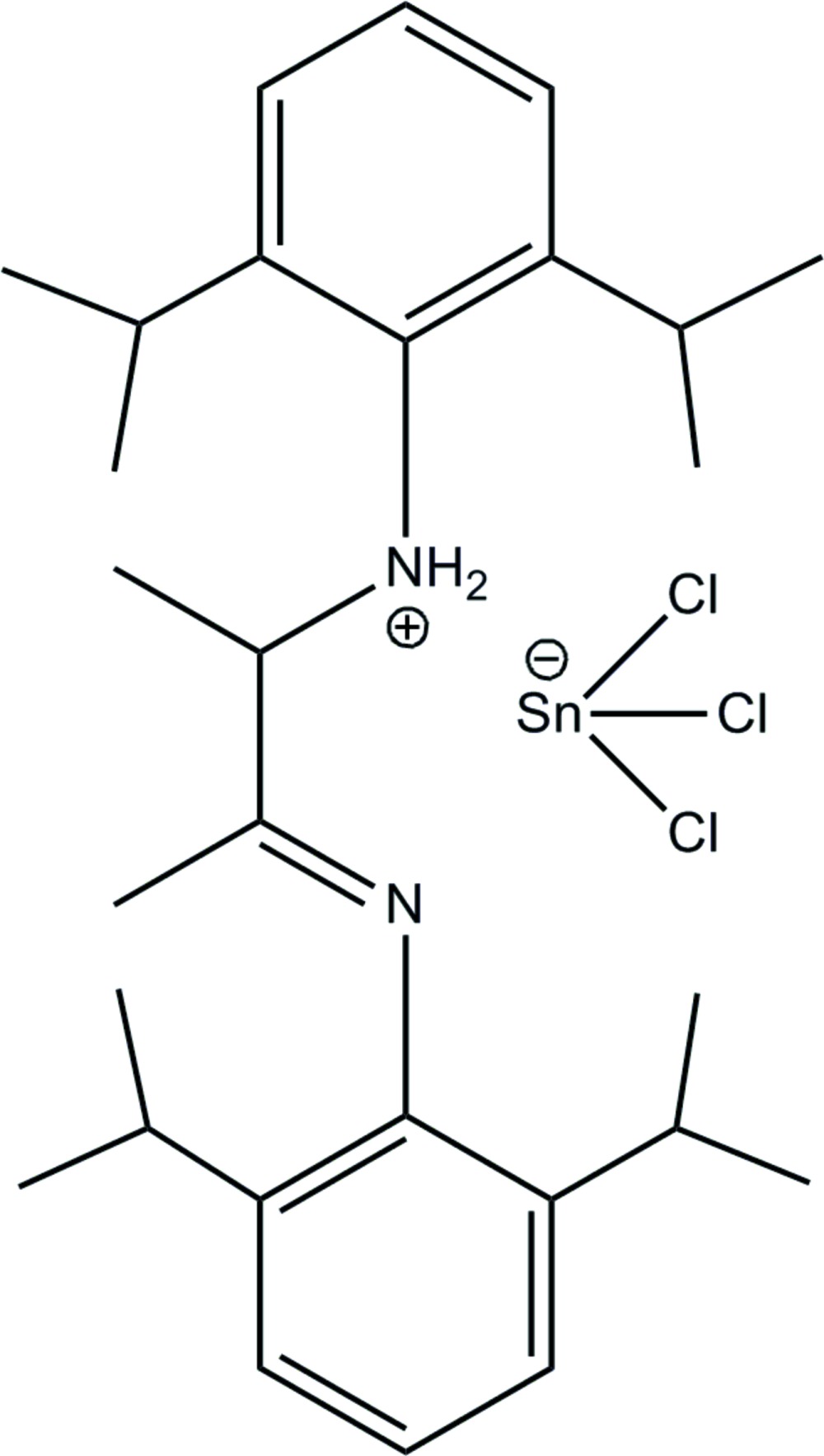


## Experimental

### 

#### Crystal data

(C_28_H_43_Cl_3_N_2_)[SnCl_3_]
*M* *_r_* = 632.68
Triclinic, 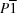

*a* = 13.373 (3) Å
*b* = 13.383 (3) Å
*c* = 18.303 (4) Å
α = 89.31 (3)°
β = 88.73 (3)°
γ = 73.67 (3)°
*V* = 3142.7 (12) Å^3^
*Z* = 4
Mo *K*α radiation
μ = 1.09 mm^−1^
*T* = 153 K
0.27 × 0.19 × 0.06 mm

#### Data collection

Rigaku AFC10/Saturn724+ diffractometer
Absorption correction: multi-scan (*CrystalClear*; Rigaku, 2007 ▶) *T*_min_ = 0.758, *T*_max_ = 0.938
26733 measured reflections
11362 independent reflections
8991 reflections with *I* > 2σ(*I*)
*R*_int_ = 0.039

#### Refinement

*R*[*F* ^2^ > 2σ(*F* ^2^)] = 0.039
*wR*(*F* ^2^) = 0.085
*S* = 1.05
11362 reflections
646 parameters
H atoms treated by a mixture of independent and constrained refinement
Δρ_max_ = 0.73 e Å^−3^
Δρ_min_ = −0.40 e Å^−3^

### 

Data collection: *CrystalClear* (Rigaku, 2008 ▶); cell refinement: *CrystalClear*; data reduction: *CrystalClear*; program(s) used to solve structure: *SHELXS97* (Sheldrick, 2008 ▶); program(s) used to refine structure: *SHELXL97* (Sheldrick, 2008 ▶); molecular graphics: *SHELXTL* (Sheldrick, 2008 ▶); software used to prepare material for publication: *SHELXTL*.

## Supplementary Material

Crystal structure: contains datablock(s) I, hp2032. DOI: 10.1107/S1600536812014729/hp2032sup1.cif

Structure factors: contains datablock(s) I. DOI: 10.1107/S1600536812014729/hp2032Isup2.hkl

Additional supplementary materials:  crystallographic information; 3D view; checkCIF report

## Figures and Tables

**Table:** 

Sn1—Cl1	2.4498 (12)
Sn1—Cl2	2.4824 (13)
Sn1—Cl3	2.4959 (13)
Sn2—Cl4	2.4647 (14)
Sn2—Cl5	2.4949 (13)
Sn2—Cl6	2.5001 (12)

**Table:** 

Cl1—Sn1—Cl2	94.54 (5)
Cl1—Sn1—Cl3	92.63 (5)
Cl2—Sn1—Cl3	91.06 (4)
Cl4—Sn2—Cl5	95.23 (5)
Cl4—Sn2—Cl6	94.23 (5)
Cl5—Sn2—Cl6	90.53 (4)

**Table 2:** Hydrogen-bond geometry (Å, °)

*D*—H⋯*A*	*D*—H	H⋯*A*	*D*⋯*A*	*D*—H⋯*A*
N2—H2*B*⋯Cl2	0.94 (4)	2.49 (4)	3.214 (3)	133 (3)
N2—H2*B*⋯Cl3	0.94 (4)	2.74 (3)	3.478 (3)	136 (3)
N4—H4*D*⋯N3	0.93 (4)	1.97 (3)	2.560 (4)	120 (3)
N2—H2*A*⋯N1	0.83 (4)	1.95 (4)	2.569 (4)	131 (3)
C11—H11⋯N2	1.00	2.50	2.917 (5)	105
C14—H14⋯N2	1.00	2.44	2.948 (5)	111
C23—H23⋯N1	1.00	2.38	2.883 (4)	110
C26—H26⋯N1	1.00	2.50	2.939 (5)	106
C39—H39⋯N4	1.00	2.46	2.951 (5)	110
C42—H42⋯N4	1.00	2.50	2.897 (5)	103
C51—H51⋯N3	1.00	2.41	2.920 (5)	111
C54—H54⋯N3	1.00	2.40	2.892 (5)	110
N4—H4*E*⋯Cl5^i^	0.90 (4)	2.38 (4)	3.194 (3)	151 (3)
C31—H31⋯Cl6^i^	1.00	2.80	3.470 (4)	125

Symmetry code: (i) 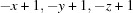.

**Fig. 1.**
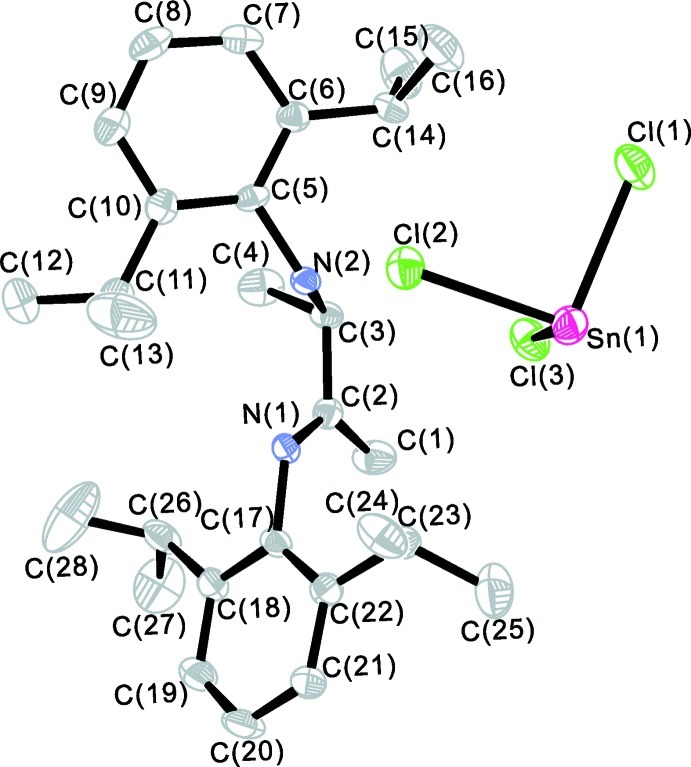
ORTEP diagram of (I) at the 50% probability

**Fig. 2.**
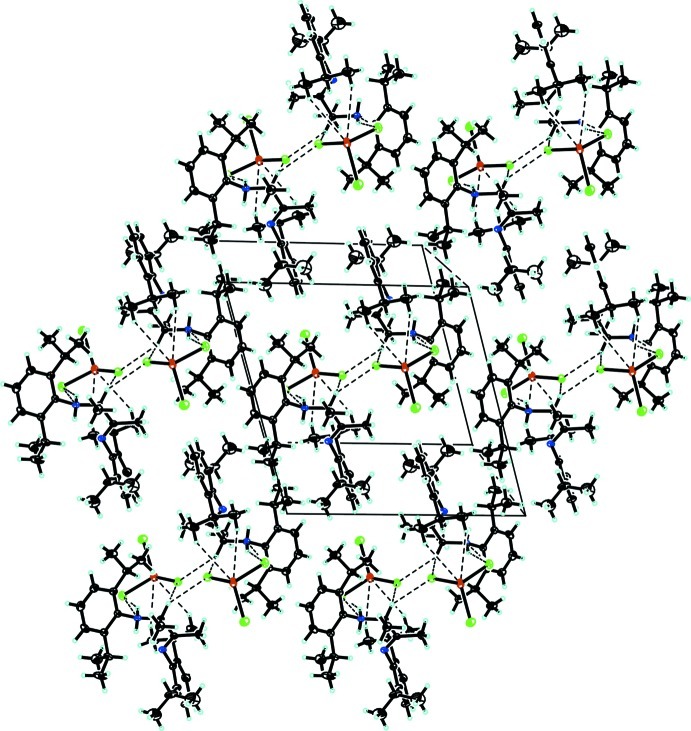
Packing diagrams showing the hydrogen bonds of (I)

## supplementary crystallographic information

### Comment

Recently, complexes supported by α-diimine ligands have attracted considerable interest (Liu *et al.*, 2011). The steric and electronic properties of such ligands can be readily modified by attaching variable substituents on the carbon and nitrogen atoms of the NCCN backbone. In addition, as redox-active ligands, they can take one or two electrons to form the mono- and dianionic species upon reduction, which makes them particularly useful in the synthesis of subvalent metal complexes. On the other hand, tin chloride complexes with lithium salt of diimine ligands have been evidenced to be a reactive species (Rake *et al.*, 2001). Herein, we report on a novel ionic complex which was synthesized by the reaction of [C(Me)NAr]_2_Li with tintetrachloride in THF at room temperature.

The molecular species of the title compound, (C_28_H_43_N_2_)[SnCl_3_] is shown in Fig. 1.

### Experimental

All manipulations were carried out under an argon atmosphere using standard Schlenk techniques. Hexane and THF was dried over sodium and freshly distilled prior to use. [(2,6-iPr_2_C_6_H_3_)-NC(Me)]_2_(404 mg, 1 mmol) was dissolved in THF (10 ml) with lithium powder (40 mg, 4 mmol) added. The resultant suspension was stirred at room temperature for three days to give an red suspension. Then it was filtered and the filtrate added to a solution of SnCl_4_ (0.12 ml, 1.0 mmol) in THF (10 ml) at 273 K over 5 min. The resultant solution was warmed to room temperature and stirred for 16 h.

Volatiles were removed *in vacuo*. The residue was extracted by hexane (10 ml), and the extract was placed at 275 K to give colorless crystals overnight.

### Refinement

C—H were included in the riding model approximation with C—H distances 0.95–0.98 Å, and with *U*_iso_(H) = 1.2*U*_eq_(C) or 1.5*U*_eq_(*C*)(methyl). H atoms that bond with N_2_ are obtained by difference Fourier method, and the *X*, Y, *Z*, *U*_eq_ take part in the minimum correction.

### Crystal data

**Table:**

(C_28_H_43_Cl_3_N_2_)[SnCl_3_]	*Z* = 4
*M**_r_* = 632.68	*F*(000) = 1304
Triclinic, *P*1	*D*_x_ = 1.337 Mg m^−^^3^
*a* = 13.373 (3) Å	Mo *K*α radiation, λ = 0.71073 Å
*b* = 13.383 (3) Å	Cell parameters from 8932 reflections
*c* = 18.303 (4) Å	θ = 2.5–29.1°
α = 89.31 (3)°	µ = 1.09 mm^−^^1^
β = 88.73 (3)°	*T* = 153 K
γ = 73.67 (3)°	Platelet, colorless
*V* = 3142.7 (12) Å^3^	0.27 × 0.19 × 0.06 mm

### Data collection

**Table:**

Rigaku AFC10/Saturn724+ diffractometer	11362 independent reflections
Radiation source: Rotating Anode	8991 reflections with *I* > 2σ(*I*)
Graphite monochromator	*R*_int_ = 0.039
Detector resolution: 28.5714 pixels mm^-1^	θ_max_ = 25.4°, θ_min_ = 2.5°
phi and ω scans	*h* = −16→16
Absorption correction: multi-scan (*CrystalClear*; Rigaku, 2007)	*k* = −16→16
*T*_min_ = 0.758, *T*_max_ = 0.938	*l* = −19→22
26733 measured reflections	

### Refinement

**Table:**

Refinement on *F*^2^	Primary atom site location: structure-invariant direct methods
Least-squares matrix: full	Secondary atom site location: difference Fourier map
*R*[*F*^2^ > 2σ(*F*^2^)] = 0.039	Hydrogen site location: inferred from neighbouring sites
*wR*(*F*^2^) = 0.085	H atoms treated by a mixture of independent and constrained refinement
*S* = 1.05	*w* = 1/[σ^2^(*F*_o_^2^) + (0.0352*P*)^2^ + 0.0722*P*] where *P* = (*F*_o_^2^ + 2*F*_c_^2^)/3
11362 reflections	(Δ/σ)_max_ = 0.001
646 parameters	Δρ_max_ = 0.73 e Å^−^^3^
0 restraints	Δρ_min_ = −0.40 e Å^−^^3^

### Special details

**Table:**

Geometry. All e.s.d.'s (except the e.s.d. in the dihedral angle between two l.s. planes) are estimated using the full covariance matrix. The cell e.s.d.'s are taken into account individually in the estimation of e.s.d.'s in distances, angles and torsion angles; correlations between e.s.d.'s in cell parameters are only used when they are defined by crystal symmetry. An approximate (isotropic) treatment of cell e.s.d.'s is used for estimating e.s.d.'s involving l.s. planes.
Refinement. Refinement of *F*^2^ against ALL reflections. The weighted *R*-factor *wR* and goodness of fit *S* are based on *F*^2^, conventional *R*-factors *R* are based on *F*, with *F* set to zero for negative *F*^2^. The threshold expression of *F*^2^ > σ(*F*^2^) is used only for calculating *R*-factors(gt) *etc*. and is not relevant to the choice of reflections for refinement. *R*-factors based on *F*^2^ are statistically about twice as large as those based on *F*, and *R*- factors based on ALL data will be even larger.

### Fractional atomic coordinates and isotropic or equivalent isotropic displacement parameters (Å^2^)

**Table:**

	*x*	*y*	*z*	*U*_iso_*/*U*_eq_	
N1	0.2928 (2)	0.3213 (2)	1.01001 (14)	0.0191 (6)	
N2	0.2250 (2)	0.1623 (2)	1.03448 (16)	0.0198 (6)	
H2A	0.210 (3)	0.222 (3)	1.0177 (19)	0.024*	
H2B	0.243 (3)	0.123 (3)	0.9913 (19)	0.024*	
C1	0.4750 (3)	0.2407 (3)	1.0461 (2)	0.0387 (10)	
H1A	0.5173	0.1758	1.0241	0.058*	
H1B	0.4915	0.2416	1.0980	0.058*	
H1C	0.4902	0.3000	1.0211	0.058*	
C2	0.3621 (3)	0.2480 (3)	1.03892 (18)	0.0230 (8)	
C3	0.3257 (3)	0.1591 (3)	1.07200 (18)	0.0222 (8)	
H3	0.3788	0.0915	1.0612	0.027*	
C4	0.3104 (3)	0.1719 (3)	1.15396 (19)	0.0375 (10)	
H4A	0.2596	0.2390	1.1646	0.056*	
H4B	0.3770	0.1697	1.1762	0.056*	
H4C	0.2847	0.1154	1.1741	0.056*	
C5	0.1411 (3)	0.1316 (3)	1.07545 (18)	0.0213 (8)	
C6	0.1508 (3)	0.0269 (3)	1.08659 (19)	0.0236 (8)	
C7	0.0696 (3)	0.0012 (3)	1.1246 (2)	0.0322 (9)	
H7	0.0733	−0.0699	1.1333	0.039*	
C8	−0.0162 (3)	0.0783 (3)	1.1497 (2)	0.0360 (10)	
H8	−0.0705	0.0599	1.1760	0.043*	
C9	−0.0232 (3)	0.1805 (3)	1.1367 (2)	0.0343 (10)	
H9	−0.0835	0.2323	1.1533	0.041*	
C10	0.0558 (3)	0.2114 (3)	1.09947 (19)	0.0266 (8)	
C11	0.0413 (3)	0.3256 (3)	1.0823 (2)	0.0388 (10)	
H11	0.1120	0.3361	1.0748	0.047*	
C12	−0.0136 (4)	0.3970 (4)	1.1434 (3)	0.0712 (18)	
H12A	−0.0177	0.4693	1.1303	0.107*	
H12B	0.0254	0.3780	1.1885	0.107*	
H12C	−0.0841	0.3900	1.1507	0.107*	
C13	−0.0170 (4)	0.3528 (4)	1.0106 (3)	0.0710 (16)	
H13A	−0.0866	0.3429	1.0165	0.107*	
H13B	0.0216	0.3074	0.9717	0.107*	
H13C	−0.0235	0.4256	0.9977	0.107*	
C14	0.2419 (3)	−0.0602 (3)	1.0577 (2)	0.0320 (9)	
H14	0.2894	−0.0276	1.0293	0.038*	
C15	0.3045 (3)	−0.1245 (4)	1.1196 (3)	0.0511 (12)	
H15A	0.3320	−0.0793	1.1503	0.077*	
H15B	0.3624	−0.1796	1.0988	0.077*	
H15C	0.2591	−0.1558	1.1492	0.077*	
C16	0.2034 (4)	−0.1287 (3)	1.0058 (2)	0.0520 (13)	
H16A	0.1611	−0.1661	1.0332	0.078*	
H16B	0.2632	−0.1790	0.9828	0.078*	
H16C	0.1610	−0.0852	0.9680	0.078*	
C17	0.3129 (3)	0.4138 (3)	0.98089 (19)	0.0230 (8)	
C18	0.3158 (3)	0.4944 (3)	1.02787 (19)	0.0268 (8)	
C19	0.3233 (3)	0.5874 (3)	0.9960 (2)	0.0346 (10)	
H19	0.3260	0.6433	1.0267	0.041*	
C20	0.3269 (3)	0.6003 (3)	0.9212 (2)	0.0372 (10)	
H20	0.3306	0.6649	0.9009	0.045*	
C21	0.3249 (3)	0.5193 (3)	0.8761 (2)	0.0298 (9)	
H21	0.3280	0.5284	0.8246	0.036*	
C22	0.3186 (3)	0.4243 (3)	0.90450 (19)	0.0243 (8)	
C23	0.3202 (3)	0.3344 (3)	0.85387 (19)	0.0274 (9)	
H23	0.3219	0.2721	0.8850	0.033*	
C24	0.2231 (3)	0.3572 (3)	0.8082 (2)	0.0439 (11)	
H24A	0.2198	0.4177	0.7766	0.066*	
H24B	0.1613	0.3720	0.8404	0.066*	
H24C	0.2255	0.2967	0.7779	0.066*	
C25	0.4176 (3)	0.3077 (4)	0.8056 (2)	0.0496 (12)	
H25A	0.4175	0.2490	0.7740	0.074*	
H25B	0.4794	0.2885	0.8362	0.074*	
H25C	0.4189	0.3682	0.7753	0.074*	
C26	0.3094 (3)	0.4850 (3)	1.1107 (2)	0.0364 (10)	
H26	0.3237	0.4094	1.1232	0.044*	
C27	0.3890 (4)	0.5257 (5)	1.1487 (3)	0.0625 (15)	
H27A	0.4577	0.4968	1.1254	0.094*	
H27B	0.3919	0.5049	1.2003	0.094*	
H27C	0.3693	0.6018	1.1450	0.094*	
C28	0.2009 (4)	0.5401 (6)	1.1394 (3)	0.087 (2)	
H28A	0.1833	0.6139	1.1257	0.130*	
H28B	0.1989	0.5337	1.1928	0.130*	
H28C	0.1504	0.5084	1.1184	0.130*	
N3	0.1739 (2)	0.7210 (2)	0.47112 (15)	0.0253 (7)	
N4	0.3336 (2)	0.7892 (2)	0.45414 (16)	0.0219 (7)	
H4D	0.264 (3)	0.815 (3)	0.4670 (18)	0.026*	
H4E	0.361 (3)	0.782 (3)	0.499 (2)	0.026*	
C29	0.2606 (3)	0.5360 (3)	0.4427 (3)	0.0482 (12)	
H29A	0.2772	0.5128	0.3922	0.072*	
H29B	0.1945	0.5236	0.4583	0.072*	
H29C	0.3163	0.4973	0.4747	0.072*	
C30	0.2512 (3)	0.6492 (3)	0.44730 (19)	0.0269 (8)	
C31	0.3449 (3)	0.6842 (3)	0.41958 (19)	0.0256 (8)	
H31	0.4105	0.6336	0.4367	0.031*	
C32	0.3468 (3)	0.6885 (3)	0.33626 (19)	0.0390 (11)	
H32A	0.4074	0.7103	0.3191	0.058*	
H32B	0.2829	0.7386	0.3193	0.058*	
H32C	0.3512	0.6194	0.3169	0.058*	
C33	0.3721 (3)	0.8670 (3)	0.41295 (17)	0.0221 (8)	
C34	0.2987 (3)	0.9464 (3)	0.37668 (19)	0.0283 (9)	
C35	0.3364 (3)	1.0177 (3)	0.3369 (2)	0.0365 (10)	
H35	0.2892	1.0735	0.3121	0.044*	
C36	0.4414 (3)	1.0083 (3)	0.3332 (2)	0.0400 (11)	
H36	0.4661	1.0564	0.3044	0.048*	
C37	0.5105 (3)	0.9311 (3)	0.3701 (2)	0.0350 (10)	
H37	0.5825	0.9273	0.3676	0.042*	
C38	0.4780 (3)	0.8575 (3)	0.41172 (19)	0.0269 (9)	
C39	0.5576 (3)	0.7762 (3)	0.4546 (2)	0.0306 (9)	
H39	0.5186	0.7389	0.4870	0.037*	
C40	0.6292 (3)	0.6954 (3)	0.4047 (2)	0.0412 (11)	
H40A	0.6813	0.6460	0.4341	0.062*	
H40B	0.6645	0.7304	0.3695	0.062*	
H40C	0.5879	0.6580	0.3784	0.062*	
C41	0.6196 (3)	0.8268 (4)	0.5034 (2)	0.0475 (12)	
H41A	0.5717	0.8814	0.5325	0.071*	
H41B	0.6649	0.8574	0.4732	0.071*	
H41C	0.6624	0.7741	0.5361	0.071*	
C42	0.1818 (3)	0.9585 (3)	0.3833 (2)	0.0345 (10)	
H42	0.1720	0.8875	0.3900	0.041*	
C43	0.1376 (4)	1.0223 (4)	0.4515 (3)	0.0597 (14)	
H43A	0.1483	1.0916	0.4469	0.089*	
H43B	0.1733	0.9870	0.4948	0.089*	
H43C	0.0628	1.0292	0.4564	0.089*	
C44	0.1224 (4)	1.0079 (5)	0.3153 (3)	0.0707 (17)	
H44A	0.1532	0.9673	0.2721	0.106*	
H44B	0.1270	1.0794	0.3095	0.106*	
H44C	0.0492	1.0087	0.3208	0.106*	
C45	0.0797 (3)	0.6984 (3)	0.4974 (2)	0.0288 (9)	
C46	0.0697 (3)	0.6795 (3)	0.5714 (2)	0.0318 (9)	
C47	−0.0238 (3)	0.6636 (3)	0.5970 (3)	0.0440 (11)	
H47	−0.0323	0.6501	0.6475	0.053*	
C48	−0.1034 (3)	0.6671 (4)	0.5504 (3)	0.0511 (13)	
H48	−0.1665	0.6558	0.5685	0.061*	
C49	−0.0918 (3)	0.6870 (4)	0.4773 (3)	0.0507 (12)	
H49	−0.1474	0.6885	0.4455	0.061*	
C50	−0.0012 (3)	0.7051 (3)	0.4484 (2)	0.0409 (11)	
C51	0.0082 (4)	0.7292 (5)	0.3680 (2)	0.0593 (15)	
H51	0.0767	0.7445	0.3599	0.071*	
C52	0.0079 (5)	0.6372 (6)	0.3193 (3)	0.103 (2)	
H52A	−0.0571	0.6183	0.3276	0.155*	
H52B	0.0672	0.5777	0.3313	0.155*	
H52C	0.0134	0.6568	0.2679	0.155*	
C53	−0.0770 (5)	0.8262 (5)	0.3458 (3)	0.085 (2)	
H53A	−0.0759	0.8841	0.3778	0.128*	
H53B	−0.1450	0.8124	0.3502	0.128*	
H53C	−0.0647	0.8447	0.2950	0.128*	
C54	0.1554 (3)	0.6767 (3)	0.6251 (2)	0.0370 (10)	
H54	0.2188	0.6797	0.5961	0.044*	
C55	0.1837 (4)	0.5760 (4)	0.6691 (3)	0.0624 (16)	
H55A	0.2029	0.5166	0.6358	0.094*	
H55B	0.1238	0.5720	0.6996	0.094*	
H55C	0.2427	0.5743	0.7004	0.094*	
C56	0.1249 (4)	0.7720 (4)	0.6742 (3)	0.0615 (14)	
H56A	0.0597	0.7743	0.7005	0.092*	
H56B	0.1154	0.8351	0.6443	0.092*	
H56C	0.1801	0.7678	0.7094	0.092*	
Sn1	0.31180 (2)	0.04625 (2)	0.801093 (14)	0.03007 (8)	
Cl1	0.31358 (9)	−0.13535 (8)	0.82219 (5)	0.0395 (3)	
Cl2	0.15041 (7)	0.11946 (8)	0.87502 (5)	0.0316 (2)	
Cl3	0.42191 (8)	0.04716 (8)	0.90928 (5)	0.0355 (2)	
Sn2	0.56678 (2)	0.31401 (2)	0.305800 (14)	0.03553 (9)	
Cl4	0.38313 (10)	0.31164 (10)	0.30677 (7)	0.0557 (3)	
Cl5	0.62937 (8)	0.15329 (8)	0.38171 (5)	0.0360 (2)	
Cl6	0.54589 (8)	0.42447 (8)	0.41740 (6)	0.0396 (3)	

### Atomic displacement parameters (Å^2^)

**Table:**

	*U*^11^	*U*^22^	*U*^33^	*U*^12^	*U*^13^	*U*^23^
N1	0.0224 (16)	0.0168 (15)	0.0169 (15)	−0.0036 (13)	−0.0009 (12)	−0.0003 (11)
N2	0.0233 (16)	0.0176 (16)	0.0200 (16)	−0.0080 (14)	−0.0001 (12)	−0.0008 (13)
C1	0.027 (2)	0.036 (3)	0.054 (3)	−0.0110 (19)	−0.0061 (19)	0.011 (2)
C2	0.0220 (19)	0.024 (2)	0.0232 (19)	−0.0069 (16)	−0.0001 (15)	−0.0024 (15)
C3	0.0228 (19)	0.0189 (19)	0.025 (2)	−0.0062 (15)	−0.0035 (15)	0.0053 (14)
C4	0.049 (3)	0.048 (3)	0.022 (2)	−0.022 (2)	−0.0114 (18)	0.0068 (18)
C5	0.0203 (19)	0.027 (2)	0.0210 (18)	−0.0134 (16)	−0.0010 (14)	0.0013 (15)
C6	0.025 (2)	0.024 (2)	0.0237 (19)	−0.0102 (16)	−0.0012 (15)	−0.0005 (15)
C7	0.040 (2)	0.028 (2)	0.035 (2)	−0.0191 (19)	−0.0028 (18)	0.0049 (17)
C8	0.029 (2)	0.049 (3)	0.036 (2)	−0.023 (2)	0.0043 (18)	0.0012 (19)
C9	0.025 (2)	0.037 (2)	0.039 (2)	−0.0077 (18)	0.0060 (17)	−0.0010 (18)
C10	0.024 (2)	0.028 (2)	0.027 (2)	−0.0064 (17)	0.0003 (16)	−0.0006 (16)
C11	0.026 (2)	0.030 (2)	0.059 (3)	−0.0067 (18)	0.013 (2)	−0.003 (2)
C12	0.066 (4)	0.033 (3)	0.110 (5)	−0.010 (3)	0.042 (3)	−0.018 (3)
C13	0.072 (4)	0.045 (3)	0.096 (4)	−0.017 (3)	−0.026 (3)	0.033 (3)
C14	0.040 (2)	0.018 (2)	0.038 (2)	−0.0094 (18)	0.0069 (18)	0.0002 (16)
C15	0.042 (3)	0.040 (3)	0.062 (3)	0.003 (2)	−0.010 (2)	−0.007 (2)
C16	0.082 (4)	0.029 (3)	0.044 (3)	−0.014 (2)	0.000 (2)	−0.007 (2)
C17	0.0173 (18)	0.0172 (19)	0.035 (2)	−0.0048 (15)	−0.0013 (15)	0.0010 (15)
C18	0.027 (2)	0.021 (2)	0.031 (2)	−0.0055 (16)	0.0009 (16)	−0.0023 (16)
C19	0.046 (3)	0.023 (2)	0.037 (2)	−0.0133 (19)	0.0020 (19)	−0.0032 (17)
C20	0.044 (3)	0.020 (2)	0.050 (3)	−0.0136 (19)	0.001 (2)	0.0064 (18)
C21	0.035 (2)	0.023 (2)	0.029 (2)	−0.0048 (17)	0.0038 (17)	0.0051 (16)
C22	0.0219 (19)	0.023 (2)	0.026 (2)	−0.0029 (16)	−0.0013 (15)	0.0039 (15)
C23	0.035 (2)	0.025 (2)	0.022 (2)	−0.0091 (17)	0.0017 (16)	0.0022 (15)
C24	0.051 (3)	0.028 (2)	0.055 (3)	−0.014 (2)	−0.011 (2)	−0.005 (2)
C25	0.043 (3)	0.052 (3)	0.051 (3)	−0.010 (2)	0.006 (2)	−0.021 (2)
C26	0.058 (3)	0.022 (2)	0.031 (2)	−0.014 (2)	−0.001 (2)	−0.0053 (17)
C27	0.056 (3)	0.090 (4)	0.043 (3)	−0.022 (3)	−0.015 (2)	−0.004 (3)
C28	0.063 (4)	0.166 (7)	0.032 (3)	−0.034 (4)	0.011 (3)	−0.001 (3)
N3	0.0205 (16)	0.0298 (18)	0.0243 (17)	−0.0050 (14)	−0.0023 (13)	0.0023 (13)
N4	0.0187 (16)	0.0280 (18)	0.0185 (16)	−0.0056 (14)	−0.0002 (13)	−0.0027 (13)
C29	0.031 (2)	0.033 (3)	0.078 (3)	−0.006 (2)	0.007 (2)	−0.009 (2)
C30	0.0202 (19)	0.030 (2)	0.029 (2)	−0.0040 (17)	−0.0051 (15)	−0.0018 (16)
C31	0.0203 (19)	0.024 (2)	0.032 (2)	−0.0044 (16)	−0.0041 (15)	−0.0032 (16)
C32	0.046 (3)	0.047 (3)	0.026 (2)	−0.015 (2)	0.0031 (18)	−0.0132 (19)
C33	0.026 (2)	0.026 (2)	0.0135 (18)	−0.0061 (16)	0.0020 (14)	0.0010 (14)
C34	0.029 (2)	0.029 (2)	0.025 (2)	−0.0061 (17)	0.0006 (16)	0.0004 (16)
C35	0.038 (2)	0.033 (2)	0.030 (2)	0.0027 (19)	−0.0005 (18)	0.0074 (18)
C36	0.050 (3)	0.043 (3)	0.032 (2)	−0.021 (2)	0.006 (2)	0.0045 (19)
C37	0.032 (2)	0.045 (3)	0.030 (2)	−0.016 (2)	0.0067 (18)	0.0014 (19)
C38	0.025 (2)	0.033 (2)	0.022 (2)	−0.0077 (17)	0.0020 (15)	−0.0002 (16)
C39	0.020 (2)	0.043 (3)	0.029 (2)	−0.0099 (18)	−0.0005 (16)	0.0068 (17)
C40	0.035 (2)	0.041 (3)	0.047 (3)	−0.009 (2)	−0.0041 (19)	−0.004 (2)
C41	0.038 (3)	0.069 (3)	0.032 (2)	−0.008 (2)	−0.0073 (19)	−0.008 (2)
C42	0.026 (2)	0.037 (2)	0.034 (2)	0.0024 (18)	−0.0051 (17)	0.0041 (18)
C43	0.043 (3)	0.068 (4)	0.063 (3)	−0.008 (3)	0.019 (2)	−0.015 (3)
C44	0.036 (3)	0.100 (5)	0.064 (4)	0.000 (3)	−0.015 (2)	0.029 (3)
C45	0.021 (2)	0.028 (2)	0.039 (2)	−0.0096 (17)	−0.0029 (16)	0.0004 (17)
C46	0.023 (2)	0.027 (2)	0.043 (2)	−0.0050 (17)	0.0016 (17)	0.0076 (17)
C47	0.034 (3)	0.044 (3)	0.052 (3)	−0.009 (2)	0.004 (2)	0.012 (2)
C48	0.026 (2)	0.046 (3)	0.084 (4)	−0.016 (2)	0.004 (2)	0.008 (3)
C49	0.028 (2)	0.055 (3)	0.072 (4)	−0.015 (2)	−0.010 (2)	−0.006 (3)
C50	0.031 (2)	0.046 (3)	0.047 (3)	−0.013 (2)	−0.0053 (19)	−0.007 (2)
C51	0.037 (3)	0.099 (5)	0.046 (3)	−0.024 (3)	−0.013 (2)	−0.005 (3)
C52	0.107 (6)	0.126 (7)	0.065 (4)	−0.012 (5)	−0.009 (4)	−0.034 (4)
C53	0.088 (5)	0.112 (6)	0.053 (4)	−0.024 (4)	−0.018 (3)	0.023 (3)
C54	0.022 (2)	0.047 (3)	0.038 (2)	−0.0047 (19)	0.0019 (17)	0.0096 (19)
C55	0.039 (3)	0.081 (4)	0.058 (3)	−0.002 (3)	−0.004 (2)	0.037 (3)
C56	0.042 (3)	0.079 (4)	0.058 (3)	−0.007 (3)	−0.009 (2)	−0.014 (3)
Sn1	0.03446 (16)	0.02990 (16)	0.02429 (15)	−0.00683 (12)	0.00233 (11)	0.00216 (11)
Cl1	0.0544 (7)	0.0267 (5)	0.0357 (6)	−0.0082 (5)	−0.0009 (5)	−0.0073 (4)
Cl2	0.0278 (5)	0.0343 (6)	0.0322 (5)	−0.0078 (4)	−0.0010 (4)	−0.0046 (4)
Cl3	0.0310 (5)	0.0292 (5)	0.0438 (6)	−0.0036 (4)	−0.0073 (4)	−0.0031 (4)
Sn2	0.0515 (2)	0.02828 (16)	0.02560 (15)	−0.00912 (14)	0.00069 (12)	−0.00350 (11)
Cl4	0.0585 (8)	0.0537 (8)	0.0571 (7)	−0.0174 (6)	−0.0257 (6)	−0.0044 (6)
Cl5	0.0477 (6)	0.0322 (6)	0.0275 (5)	−0.0101 (5)	−0.0022 (4)	−0.0011 (4)
Cl6	0.0354 (6)	0.0382 (6)	0.0432 (6)	−0.0063 (5)	−0.0001 (5)	−0.0188 (5)

### Geometric parameters (Å, º)

**Table:**

N1—C2	1.264 (4)	N4—C31	1.516 (4)
N1—C17	1.433 (4)	N4—H4D	0.93 (4)
N2—C5	1.484 (4)	N4—H4E	0.90 (4)
N2—C3	1.516 (4)	C29—C30	1.486 (5)
N2—H2A	0.83 (4)	C29—H29A	0.9800
N2—H2B	0.94 (4)	C29—H29B	0.9800
C1—C2	1.494 (5)	C29—H29C	0.9800
C1—H1A	0.9800	C30—C31	1.531 (5)
C1—H1B	0.9800	C31—C32	1.525 (5)
C1—H1C	0.9800	C31—H31	1.0000
C2—C3	1.521 (5)	C32—H32A	0.9800
C3—C4	1.515 (5)	C32—H32B	0.9800
C3—H3	1.0000	C32—H32C	0.9800
C4—H4A	0.9800	C33—C38	1.385 (5)
C4—H4B	0.9800	C33—C34	1.400 (5)
C4—H4C	0.9800	C34—C35	1.391 (5)
C5—C6	1.383 (5)	C34—C42	1.528 (5)
C5—C10	1.392 (5)	C35—C36	1.374 (6)
C6—C7	1.398 (5)	C35—H35	0.9500
C6—C14	1.519 (5)	C36—C37	1.361 (6)
C7—C8	1.384 (5)	C36—H36	0.9500
C7—H7	0.9500	C37—C38	1.395 (5)
C8—C9	1.364 (6)	C37—H37	0.9500
C8—H8	0.9500	C38—C39	1.517 (5)
C9—C10	1.399 (5)	C39—C41	1.519 (5)
C9—H9	0.9500	C39—C40	1.525 (5)
C10—C11	1.516 (5)	C39—H39	1.0000
C11—C12	1.515 (6)	C40—H40A	0.9800
C11—C13	1.529 (6)	C40—H40B	0.9800
C11—H11	1.0000	C40—H40C	0.9800
C12—H12A	0.9800	C41—H41A	0.9800
C12—H12B	0.9800	C41—H41B	0.9800
C12—H12C	0.9800	C41—H41C	0.9800
C13—H13A	0.9800	C42—C43	1.531 (5)
C13—H13B	0.9800	C42—C44	1.532 (6)
C13—H13C	0.9800	C42—H42	1.0000
C14—C16	1.523 (5)	C43—H43A	0.9800
C14—C15	1.528 (6)	C43—H43B	0.9800
C14—H14	1.0000	C43—H43C	0.9800
C15—H15A	0.9800	C44—H44A	0.9800
C15—H15B	0.9800	C44—H44B	0.9800
C15—H15C	0.9800	C44—H44C	0.9800
C16—H16A	0.9800	C45—C46	1.385 (5)
C16—H16B	0.9800	C45—C50	1.403 (5)
C16—H16C	0.9800	C46—C47	1.397 (5)
C17—C18	1.398 (5)	C46—C54	1.518 (5)
C17—C22	1.406 (5)	C47—C48	1.369 (6)
C18—C19	1.396 (5)	C47—H47	0.9500
C18—C26	1.523 (5)	C48—C49	1.375 (6)
C19—C20	1.379 (5)	C48—H48	0.9500
C19—H19	0.9500	C49—C50	1.393 (6)
C20—C21	1.378 (5)	C49—H49	0.9500
C20—H20	0.9500	C50—C51	1.514 (6)
C21—C22	1.391 (5)	C51—C53	1.524 (7)
C21—H21	0.9500	C51—C52	1.529 (8)
C22—C23	1.523 (5)	C51—H51	1.0000
C23—C24	1.515 (5)	C52—H52A	0.9800
C23—C25	1.517 (5)	C52—H52B	0.9800
C23—H23	1.0000	C52—H52C	0.9800
C24—H24A	0.9800	C53—H53A	0.9800
C24—H24B	0.9800	C53—H53B	0.9800
C24—H24C	0.9800	C53—H53C	0.9800
C25—H25A	0.9800	C54—C55	1.521 (6)
C25—H25B	0.9800	C54—C56	1.523 (6)
C25—H25C	0.9800	C54—H54	1.0000
C26—C27	1.511 (6)	C55—H55A	0.9800
C26—C28	1.516 (7)	C55—H55B	0.9800
C26—H26	1.0000	C55—H55C	0.9800
C27—H27A	0.9800	C56—H56A	0.9800
C27—H27B	0.9800	C56—H56B	0.9800
C27—H27C	0.9800	C56—H56C	0.9800
C28—H28A	0.9800	Sn1—Cl1	2.4498 (12)
C28—H28B	0.9800	Sn1—Cl2	2.4824 (13)
C28—H28C	0.9800	Sn1—Cl3	2.4959 (13)
N3—C30	1.271 (4)	Sn2—Cl4	2.4647 (14)
N3—C45	1.447 (4)	Sn2—Cl5	2.4949 (13)
N4—C33	1.477 (4)	Sn2—Cl6	2.5001 (12)
			
C2—N1—C17	123.2 (3)	C31—N4—H4D	106 (2)
C5—N2—C3	119.9 (3)	C33—N4—H4E	109 (2)
C5—N2—H2A	117 (2)	C31—N4—H4E	111 (2)
C3—N2—H2A	100 (3)	H4D—N4—H4E	99 (3)
C5—N2—H2B	111 (2)	C30—C29—H29A	109.5
C3—N2—H2B	107 (2)	C30—C29—H29B	109.5
H2A—N2—H2B	101 (3)	H29A—C29—H29B	109.5
C2—C1—H1A	109.5	C30—C29—H29C	109.5
C2—C1—H1B	109.5	H29A—C29—H29C	109.5
H1A—C1—H1B	109.5	H29B—C29—H29C	109.5
C2—C1—H1C	109.5	N3—C30—C29	127.6 (3)
H1A—C1—H1C	109.5	N3—C30—C31	115.7 (3)
H1B—C1—H1C	109.5	C29—C30—C31	116.7 (3)
N1—C2—C1	127.2 (3)	N4—C31—C32	112.9 (3)
N1—C2—C3	116.3 (3)	N4—C31—C30	106.0 (3)
C1—C2—C3	116.5 (3)	C32—C31—C30	110.1 (3)
C4—C3—N2	111.9 (3)	N4—C31—H31	109.3
C4—C3—C2	110.6 (3)	C32—C31—H31	109.3
N2—C3—C2	106.0 (3)	C30—C31—H31	109.3
C4—C3—H3	109.4	C31—C32—H32A	109.5
N2—C3—H3	109.4	C31—C32—H32B	109.5
C2—C3—H3	109.4	H32A—C32—H32B	109.5
C3—C4—H4A	109.5	C31—C32—H32C	109.5
C3—C4—H4B	109.5	H32A—C32—H32C	109.5
H4A—C4—H4B	109.5	H32B—C32—H32C	109.5
C3—C4—H4C	109.5	C38—C33—C34	123.5 (3)
H4A—C4—H4C	109.5	C38—C33—N4	118.9 (3)
H4B—C4—H4C	109.5	C34—C33—N4	117.6 (3)
C6—C5—C10	123.9 (3)	C35—C34—C33	116.9 (3)
C6—C5—N2	118.9 (3)	C35—C34—C42	121.0 (4)
C10—C5—N2	117.2 (3)	C33—C34—C42	122.1 (3)
C5—C6—C7	117.2 (3)	C36—C35—C34	120.7 (4)
C5—C6—C14	123.9 (3)	C36—C35—H35	119.7
C7—C6—C14	118.9 (3)	C34—C35—H35	119.7
C8—C7—C6	120.6 (4)	C37—C36—C35	120.8 (4)
C8—C7—H7	119.7	C37—C36—H36	119.6
C6—C7—H7	119.7	C35—C36—H36	119.6
C9—C8—C7	120.3 (3)	C36—C37—C38	121.5 (4)
C9—C8—H8	119.9	C36—C37—H37	119.2
C7—C8—H8	119.9	C38—C37—H37	119.2
C8—C9—C10	121.9 (4)	C33—C38—C37	116.5 (4)
C8—C9—H9	119.1	C33—C38—C39	124.2 (3)
C10—C9—H9	119.1	C37—C38—C39	119.3 (3)
C5—C10—C9	116.2 (3)	C38—C39—C41	111.1 (4)
C5—C10—C11	123.9 (3)	C38—C39—C40	111.8 (3)
C9—C10—C11	119.8 (3)	C41—C39—C40	111.3 (3)
C12—C11—C10	113.1 (3)	C38—C39—H39	107.5
C12—C11—C13	111.2 (4)	C41—C39—H39	107.5
C10—C11—C13	108.9 (4)	C40—C39—H39	107.5
C12—C11—H11	107.8	C39—C40—H40A	109.5
C10—C11—H11	107.8	C39—C40—H40B	109.5
C13—C11—H11	107.8	H40A—C40—H40B	109.5
C11—C12—H12A	109.5	C39—C40—H40C	109.5
C11—C12—H12B	109.5	H40A—C40—H40C	109.5
H12A—C12—H12B	109.5	H40B—C40—H40C	109.5
C11—C12—H12C	109.5	C39—C41—H41A	109.5
H12A—C12—H12C	109.5	C39—C41—H41B	109.5
H12B—C12—H12C	109.5	H41A—C41—H41B	109.5
C11—C13—H13A	109.5	C39—C41—H41C	109.5
C11—C13—H13B	109.5	H41A—C41—H41C	109.5
H13A—C13—H13B	109.5	H41B—C41—H41C	109.5
C11—C13—H13C	109.5	C34—C42—C43	109.2 (3)
H13A—C13—H13C	109.5	C34—C42—C44	112.8 (3)
H13B—C13—H13C	109.5	C43—C42—C44	110.7 (4)
C6—C14—C16	110.2 (3)	C34—C42—H42	108.0
C6—C14—C15	111.9 (3)	C43—C42—H42	108.0
C16—C14—C15	111.2 (4)	C44—C42—H42	108.0
C6—C14—H14	107.8	C42—C43—H43A	109.5
C16—C14—H14	107.8	C42—C43—H43B	109.5
C15—C14—H14	107.8	H43A—C43—H43B	109.5
C14—C15—H15A	109.5	C42—C43—H43C	109.5
C14—C15—H15B	109.5	H43A—C43—H43C	109.5
H15A—C15—H15B	109.5	H43B—C43—H43C	109.5
C14—C15—H15C	109.5	C42—C44—H44A	109.5
H15A—C15—H15C	109.5	C42—C44—H44B	109.5
H15B—C15—H15C	109.5	H44A—C44—H44B	109.5
C14—C16—H16A	109.5	C42—C44—H44C	109.5
C14—C16—H16B	109.5	H44A—C44—H44C	109.5
H16A—C16—H16B	109.5	H44B—C44—H44C	109.5
C14—C16—H16C	109.5	C46—C45—C50	122.5 (3)
H16A—C16—H16C	109.5	C46—C45—N3	118.4 (3)
H16B—C16—H16C	109.5	C50—C45—N3	118.9 (3)
C18—C17—C22	121.9 (3)	C45—C46—C47	118.0 (4)
C18—C17—N1	119.7 (3)	C45—C46—C54	122.6 (3)
C22—C17—N1	118.0 (3)	C47—C46—C54	119.3 (4)
C19—C18—C17	117.4 (3)	C48—C47—C46	120.9 (4)
C19—C18—C26	119.8 (3)	C48—C47—H47	119.5
C17—C18—C26	122.9 (3)	C46—C47—H47	119.5
C20—C19—C18	121.8 (4)	C47—C48—C49	119.9 (4)
C20—C19—H19	119.1	C47—C48—H48	120.1
C18—C19—H19	119.1	C49—C48—H48	120.1
C21—C20—C19	119.7 (4)	C48—C49—C50	122.1 (4)
C21—C20—H20	120.1	C48—C49—H49	119.0
C19—C20—H20	120.1	C50—C49—H49	119.0
C20—C21—C22	121.2 (4)	C49—C50—C45	116.5 (4)
C20—C21—H21	119.4	C49—C50—C51	120.8 (4)
C22—C21—H21	119.4	C45—C50—C51	122.6 (4)
C21—C22—C17	118.0 (3)	C50—C51—C53	111.0 (4)
C21—C22—C23	120.5 (3)	C50—C51—C52	112.5 (5)
C17—C22—C23	121.5 (3)	C53—C51—C52	110.7 (5)
C24—C23—C25	110.8 (3)	C50—C51—H51	107.5
C24—C23—C22	111.8 (3)	C53—C51—H51	107.5
C25—C23—C22	110.9 (3)	C52—C51—H51	107.5
C24—C23—H23	107.7	C51—C52—H52A	109.5
C25—C23—H23	107.7	C51—C52—H52B	109.5
C22—C23—H23	107.7	H52A—C52—H52B	109.5
C23—C24—H24A	109.5	C51—C52—H52C	109.5
C23—C24—H24B	109.5	H52A—C52—H52C	109.5
H24A—C24—H24B	109.5	H52B—C52—H52C	109.5
C23—C24—H24C	109.5	C51—C53—H53A	109.5
H24A—C24—H24C	109.5	C51—C53—H53B	109.5
H24B—C24—H24C	109.5	H53A—C53—H53B	109.5
C23—C25—H25A	109.5	C51—C53—H53C	109.5
C23—C25—H25B	109.5	H53A—C53—H53C	109.5
H25A—C25—H25B	109.5	H53B—C53—H53C	109.5
C23—C25—H25C	109.5	C46—C54—C55	111.6 (4)
H25A—C25—H25C	109.5	C46—C54—C56	110.6 (3)
H25B—C25—H25C	109.5	C55—C54—C56	111.8 (4)
C27—C26—C28	109.9 (4)	C46—C54—H54	107.5
C27—C26—C18	112.7 (3)	C55—C54—H54	107.5
C28—C26—C18	110.9 (4)	C56—C54—H54	107.5
C27—C26—H26	107.7	C54—C55—H55A	109.5
C28—C26—H26	107.7	C54—C55—H55B	109.5
C18—C26—H26	107.7	H55A—C55—H55B	109.5
C26—C27—H27A	109.5	C54—C55—H55C	109.5
C26—C27—H27B	109.5	H55A—C55—H55C	109.5
H27A—C27—H27B	109.5	H55B—C55—H55C	109.5
C26—C27—H27C	109.5	C54—C56—H56A	109.5
H27A—C27—H27C	109.5	C54—C56—H56B	109.5
H27B—C27—H27C	109.5	H56A—C56—H56B	109.5
C26—C28—H28A	109.5	C54—C56—H56C	109.5
C26—C28—H28B	109.5	H56A—C56—H56C	109.5
H28A—C28—H28B	109.5	H56B—C56—H56C	109.5
C26—C28—H28C	109.5	Cl1—Sn1—Cl2	94.54 (5)
H28A—C28—H28C	109.5	Cl1—Sn1—Cl3	92.63 (5)
H28B—C28—H28C	109.5	Cl2—Sn1—Cl3	91.06 (4)
C30—N3—C45	121.1 (3)	Cl4—Sn2—Cl5	95.23 (5)
C33—N4—C31	118.6 (3)	Cl4—Sn2—Cl6	94.23 (5)
C33—N4—H4D	112 (2)	Cl5—Sn2—Cl6	90.53 (4)

### Hydrogen-bond geometry (Å, º)

**Table:**

*D*—H···*A*	*D*—H	H···*A*	*D*···*A*	*D*—H···*A*
N2—H2*B*···Cl2	0.94 (4)	2.49 (4)	3.214 (3)	133 (3)
N2—H2*B*···Cl3	0.94 (4)	2.74 (3)	3.478 (3)	136 (3)
N4—H4*D*···N3	0.93 (4)	1.97 (3)	2.560 (4)	120 (3)
N2—H2*A*···N1	0.83 (4)	1.95 (4)	2.569 (4)	131 (3)
C11—H11···N2	1.00	2.50	2.917 (5)	105
C14—H14···N2	1.00	2.44	2.948 (5)	111
C23—H23···N1	1.00	2.38	2.883 (4)	110
C26—H26···N1	1.00	2.50	2.939 (5)	106
C39—H39···N4	1.00	2.46	2.951 (5)	110
C42—H42···N4	1.00	2.50	2.897 (5)	103
C51—H51···N3	1.00	2.41	2.920 (5)	111
C54—H54···N3	1.00	2.40	2.892 (5)	110
N4—H4*E*···Cl5^i^	0.90 (4)	2.38 (4)	3.194 (3)	151 (3)
C31—H31···Cl6^i^	1.00	2.80	3.470 (4)	125

Symmetry code: (i) −*x*+1, −*y*+1, −*z*+1.
